# Differences in Candidate Gene Association between European Ancestry and African American Asthmatic Children

**DOI:** 10.1371/journal.pone.0016522

**Published:** 2011-02-28

**Authors:** Tesfaye M. Baye, Melinda Butsch Kovacic, Jocelyn M. Biagini Myers, Lisa J. Martin, Mark Lindsey, Tia L. Patterson, Hua He, Mark B. Ericksen, Jayanta Gupta, Anna M. Tsoras, Andrew Lindsley, Marc E. Rothenberg, Marsha Wills-Karp, N. Tony Eissa, Larry Borish, Gurjit K. Khurana Hershey

**Affiliations:** 1 Department of Pediatrics, Cincinnati Children's Hospital Medical Center, University of Cincinnati, Cincinnati, Ohio, United States of America; 2 Department of Medicine, Baylor College of Medicine, Houston, Texas, United States of America; 3 Department of Medicine, University of Virginia, Charlottesville, Virginia, United States of America; Albert Einstein Institute for Research and Education, Brazil

## Abstract

**Background:**

Candidate gene case-control studies have identified several single nucleotide polymorphisms (SNPs) that are associated with asthma susceptibility. Most of these studies have been restricted to evaluations of specific SNPs within a single gene and within populations from European ancestry. Recently, there is increasing interest in understanding racial differences in genetic risk associated with childhood asthma. Our aim was to compare association patterns of asthma candidate genes between children of European and African ancestry.

**Methodology/Principal Findings:**

Using a custom-designed Illumina SNP array, we genotyped 1,485 children within the Greater Cincinnati Pediatric Clinic Repository and Cincinnati Genomic Control Cohort for 259 SNPs in 28 genes and evaluated their associations with asthma. We identified 14 SNPs located in 6 genes that were significantly associated (p-values <0.05) with childhood asthma in African Americans. Among Caucasians, 13 SNPs in 5 genes were associated with childhood asthma. Two SNPs in IL4 were associated with asthma in both races (p-values <0.05). Gene-gene interaction studies identified race specific sets of genes that best discriminate between asthmatic children and non-allergic controls.

**Conclusions/Significance:**

We identified IL4 as having a role in asthma susceptibility in both African American and Caucasian children. However, while IL4 SNPs were associated with asthma in asthmatic children with European and African ancestry, the relative contributions of the most replicated asthma-associated SNPs varied by ancestry. These data provides valuable insights into the pathways that may predispose to asthma in individuals with European vs. African ancestry.

## Introduction

Asthma (MIM 600807) is a disease of chronic airway inflammation characterized by recurrent episodes of wheezing, dyspnea, chest tightness, and cough. It affects nearly 300 million individuals worldwide including 20 million adults and children in the United States [1], [2]. Approximately 5,000 asthma deaths occur in the US every year [3]. Previous studies have revealed strong familial aggregation with heritability estimates between 36 and 79%, supporting the existence of asthma susceptibility genes. Indeed, more than 120 genes have been found to be associated with asthma- or atopy-related phenotypes as reported in greater than 600 studies [4].

While many studies have evaluated the importance of genetics on asthma susceptibility, most studies employ samples from populations of European descent. Few have focused on asthma risk in African Americans, despite the fact that asthma morbidity and mortality are more prevalent in this subgroup. In the PubMed database, European populations are mentioned 5 times more often in various asthma related literature than African Americans (http://www.ncbi.nlm.nih.gov). Studies in other ethnicities, particularly African-derived populations, are valuable, because they may help localize the signals of association and because additional variants present at high frequency in African-derived populations may be absent or rare in Caucasian samples [5]. Furthermore, it is not clear whether associations with asthma found in the Caucasian samples can be consistently replicated in samples from predominantly recent African ancestry. Genetic, environmental or phenotypic heterogeneity, gene-gene and gene by environment interactions or different recombination histories between populations could all contribute to a lack of replication in African-derived populations. Genetic variants may also have different effects in different populations because of unmeasured (and perhaps unknown) environmental risk factors. Hence, the prognostic utility value of specific variants for asthma risk assessment differs across populations [6]. Given the greater genetic diversity and different linkage disequilibrium (LD) structure exhibited by African-ancestry populations, understanding genetic variation in asthma related genes in African American population could provide novel insights into the etiology of asthma.

Therefore, the objective of this study was to identify the similarities and differences in association patterns of asthma and known candidate genes between European ancestry and African American children. To accomplish this objective, we used a carefully collected cohort of children from the greater Cincinnati area as the discovery cohort and an independent replication cohort of Caucasians and publicly available dataset of African Americans.

## Materials and Methods

### Study population

The analysis included Caucasian and African American asthmatic, allergic and non-allergic children enrolled in the Greater Cincinnati Pediatric Clinic Repository (GCPCR) and Cincinnati Genomic Control Cohort (GCC) and who met the case and control definitions (outlined below). Recruitment for GCPCR began in November, 2003 and is ongoing. Children with asthma and other allergic conditions visiting the allergy/immunology, pulmonary, and dermatology outpatient specialty clinics and from the Emergency Department at CCHMC were invited to participate in the GCPCR. Non-allergic control children were recruited into GCPCR from headache, dental and orthopedic clinics as well as from the community at large using paper and online advertising media. Following written informed consent, participants were asked to provide a buccal (using a cytobrush) or saliva sample (Oragene DNA Self-Collection Kit, DNA Genotek Inc., Ottawa, ON Canada) for DNA isolation and to complete repository specific questionnaires. The GCC is an ongoing community-based cohort of over 1,020 healthy children ages 3–18 years old. In terms of race, ethnicity, gender, and socioeconomic status, participants are representative of the 7 counties that cover the Greater Cincinnati region. Participating GCC children provided a blood sample for DNA isolation at their baseline visit. For these genetic association studies, GCPCR participants aged 4 to 17 years with physician diagnosed asthma based on the ATS criteria [7] (with or without allergic rhinitis and/or atopic dermatitis), and available pulmonary function test results and/or respiratory symptom scores were included as asthmatic cases. Similar aged non-asthmatic GCPCR children with allergic rhinitis and/or atopic dermatitis or non-asthmatic GCC children who reported ever having hay fever or eczema were included as allergic children. Children from either GCPCR or the GCC were included as non-allergic controls if they reported not having any personal or family history of asthma, and not having a personal history of any allergic disorder. Written informed consent was obtained from all interested patients and their parents/guardians. The study was approved by the Cincinnati Children's Hospital Medical Center Institutional Review Board.

### Replication cohorts

The Caucasian replication population includes asthmatic children from the GCC compared to non-asthmatic adults with no family history of asthma from the Cincinnati Control Cohort (CCC). Like the GCC, the CCC is a population-based sample of 298 Caucasians (age 24–90 years) from the Greater Cincinnati/Northern Kentucky area. The African American replication populations were 42 African American trios (126 individual samples) from the Childhood Asthma Management Program (CAMP) data available from the NIH-based database of Genotypes and Phenotypes (dbGaP) (http://www.ncbi.nlm.nih.gov/gap) Formal permission for use of the dbGaP data was obtained prior to analysis. Both the Caucasian and African American replication cohorts were genotyped using Affymetrix 6.0 SNP chip.

### Candidate gene and SNP selection, and genotyping

We conducted a large-scale evaluation of candidate genes to identify common variants that influence asthma risk. A total of 28 candidate genes were selected for inclusion in a custom Illumina GoldenGate™ assay. To investigate asthma liability genes systematically, we selected 28 candidate genes. These candidates were chosen based on a high number of replications in the literature (>10) [8] and biologic relevance in the pathogenesis of asthma or allergy. The description of candidate genes including function and process terms deposited in the Gene Ontology (GO) databases (http://www.geneontology.org; [∧] accessed on June 20, 2010) are shown in Table 1.

**Table 1:** Asthma candidate genes and number of SNPs used in analyses.

Gene symbol	Gene name	Chr location	SNPs genotyped*	Gene Ontology Terms (www.geneontology.org)	
				Process	Function
ATPAF1	Apoptotic protease activating factor 1	1p33	3 (1)	protein complex assembly	
CHI3L2	chitinase3like 2	1p13	13 (9)	carbohydrate metabolic process, chitin catabolic process	cation binding, hydrolase activity
CHIA	chitinase	1p13.2	31(23)	cell wall chitin metabolic process, immune response, polysaccharide catabolic process, response to fungus, response to acid	cation binding, chitin binding, lysozyme activity, sugar binding
CLCA1	chloride channel accessory 1	1p22.3	29 (24)		
FLG	filaggrin	1q21.3	12 (6)	keratinocyte differentiation, multicellular development	calcium ion binding, structural molecule activity
IL10	interleukin 10	1q32.1	10 (9)	B cell differentiation, T-helper 2 type immune response, defense response to bacterium, immune response, inflammatory response, receptor biosynthetic process, negative regulation of interleukin12	cytokine activity, interleukin10 receptor binding, protein binding
INSIG2	insulin induced gene 2	2q14.1	9 (9)	cholesterol metabolic process, lipid metabolic process, response to sterol depletion, steroid metabolic process	protein binding
NFE2L2	nuclear factor erythroidderived 2like 2	2q31.2	8 (5)	regulation of transcription, DNA dependent transcription from RNA polymerase II promoter	sequencespecific DNA binding, transcription factor activity
ADIPOQ	adiponectin, C1Q and collagen domain containing	3q27.3	13 (10)	fatty acid betaoxidation, generation of precursor metabolites and energy, protein/glucose homeostasis, lowdensity lipoprotein particle clearance, negative regulation of inflammatory response	cytokine activity, eukaryotic cell surface binding, protein homodimerization activity
ADRB2	adrenergic, beta2, receptor, surface	5q33.1	9 (6)	Gprotein coupled receptor protein signaling pathway, receptormediated endocytosis, negative regulation of inflammatory response	beta2adrenergic receptor activity, potassium channel regulator activity, receptor activity
IL13	interleukin 13	5q31.1	7 (6)	cell motion, cellcell signaling, immune response, inflammatory response, signal transduction	cytokine activity, interleukin13 receptor binding,
IL4	interleukin 4	5q3135	10 (7)	B cell differentiation, Thelper 2 type immune response, cellular defense response, cholesterol metabolic process, regulation of immune response, positive regulation of isotype switching to IgE isotypes	cytokine activity interleukin4 receptor, binding
IL9	interleukin 9	5q31.1	5 (5)	immune response, inflammatory response positive regulation of cell proliferation positive regulation of interleukin5 biosynthetic process	cytokine activity growth factor activity cytokine receptor binding
SPINK5	serine protease inhibitor Kazal type 5	5q33.1	19 (13)	negative regulation of immune response, regulation of T cell differentiation, epithelial cell differentiation	serinetype endopeptidase inhibitor activity
TSLP	thymic stromal lymphopoietin	5q22.1	9 (9)		cytokine activity
CCL26	eotaxin3	7q11.23	10 (5)	Chemotaxis, immune response inflammatory response, signal transduction	chemokine activity
SERPINE1	serpin peptidase inhibitor, clade E, member 1	7q22.1	20 (9)	chronological cell aging fibrinolysis, regulation of angiogenesis	protease binding, protein binding, serinetype endopeptidase activity
ALOX5	arachidonate 5lipoxygenase	10q11.21	14 (13)	inflammatory response, leukotriene biosynthetic process, oxidation reduction	arachidonate 5lipoxygenase activity, calcium ion binding
SPI1	spleen focus forming virus proviral integration spi1	11p11.2	8 (7)	negative regulation of transcription from RNA polymerase II promoter, positive regulation of genespecific, transcription	protein binding, sequencespecific DNA binding
SERPINA1	serpin peptidase inhibitor, clade A (alpha1 antiproteinase, antitrypsin), member 1	14q32.1	15 (15)	acutephase response, blood coagulation	peptidase activity, peptidase inhibitor activity, protease binding, protein binding, serinetype endopeptidase inhibitor activity
CIITA	class II, major histocompatibility complex	16p13.13	13 (8)	immune response, regulation of transcription, DNAdependent, response to antibiotic	protein binding, transcription coactivator activity
IL4Rα	interleukin 4Rα	16p11	31 (16)	immune response, signal transduction	interleukin4 receptor activity protein binding, receptor activity
STUB1	STIP1 homology and Ubox protein 1	16p13.3	4 (1)	protein polyubiquitination, regulation of glucocorticoid metabolic process, ubiquitindependent SMAD protein catabolic process	Hsp70 protein binding,
HRH4	histamine receptor H4	18q11.2	14 (11)	Gprotein coupled receptor protein signaling pathway, signal transduction	histamine receptor activity,
TGFB1	transforming growth factor, beta 1	19q13.2	5 (4)	positive regulation of interleukin17 production, induction of apoptosis, inflammatory response, lymph node development	protein Nterminus binding type II transforming growth factor
CDH26	cadherinlike 26	20q13.33	12 (8)	integral to membrane, plasma membrane	homophilic cell adhesion
IL13RA1	interleukin 13R1	Xq24	17 (14)		receptor activity
IL13RA2	interleukin 13R2	Xq13	5 (4)	extracellular space, integral to membrane	cytokine receptor activity

*numbers in parentheses indicates number of SNPs that passed quality control and enter to statistical analysis.

SNPs for this chip were selected in one of two ways. First, non-synonymous SNPs or SNPs in regulatory or coding regions were selected. Second, tagging SNPs that efficiently capture all the common genetic variation in a gene were selected using Haploview and Tagger (http://www.broad.mit.edu/mpg/haploview). The rationale for tagging SNPs is that genetic variants that are near each other and in linkage disequilibrium (LD) tend to be inherited together as a result of shared ancestry. The strong correlations between markers within haplotype blocks help to enable accurate representation of a gene region by a small number of tagging SNPs. The SNPs were retrieved from Caucasians in the United States with northern and western European ancestry [CEU] and Yorubans in Ibadan, Nigeria [YRI] population samples of the public HapMap database (http://hapmap.ncbi.nlm.nih.gov). Genotyping using the Illumina GoldenGate Assay (http://www.illumina.com) system was performed at the CCHMC Genetic Variation and Gene Discovery Core. Genotypes were assigned using Illumina's BeadStudio v3.2 Software (San Diego, CA).

### Statistical analysis

All analyses were performed separately in Caucasian and African American datasets. Prior to analysis, SNPs which failed Hardy Weinberg Equilibrium (HWE) in the control dataset (p<0.0001) or had poor genotype calling (missing rate greater than 10%) or minor allele frequencies below 10% were excluded from the analysis. In addition, individuals with more than 20% of their total SNPs missing were also removed from the analysis. To account for potential population stratification/confounding or admixture in these samples, principal component analyses (PCA) was performed using 30 unlinked Ancestry Informative Markers (AIMs) and the EIGENSTRAT software [9]. The principal component score for each individual was included as a covariate in all models along with age and gender in logistic regression models.

Statistical comparisons in both Caucasians and African Americans were made between asthmatic children and non-allergic controls and also between the allergic children and the non-allergic controls. As a general association screen, we tested for the additive models of single SNP analysis, which assume that each copy of the risk allele will increase disease prevalence. Unconditional logistic regression was used to calculate p-values and odds ratios for each SNP using the software PLINK (V1.05) and Bonferroni adjustment that scales the original threshold by the number of tests performed was used to correct for multiple testing and determine the statistical significance of each SNP [10]. To investigate the relationship between IL4 and IL13 genes, linkage disequilibrium (LD) plots were computed independently for Caucasians and African Americans using Haploview version 4.1 [11]. Results were evaluated after correcting for multiple testing using a Bonferroni adjustment taking into account LD correlation between SNPs.

To compare the allele frequencies between Caucasian and African Americans asthmatic and non-allergic controls, we used the absolute allele frequency difference also called delta (δ). It is defined as the absolute value of the difference of the frequency of a particular allele observed between the two populations. If we let P_11_ represent the frequency of allele in the first population and P_21_ the frequency of the same allele in the second population, then δ = |P_11_−P_21_|. A marker with δ = 1 provides perfect information regarding ancestry whereas a marker with δ = 0 carries no information [12].

Recursive partitioning (RP) was used to evaluate gene-gene interactions using the R package PARTY (v0.9-995; www.r-project.org). The purpose of RP is to identify the optimum combination of SNPs that best discriminate between asthmatic and control subjects. RP uses a series of regressions to identify covariates (in this case SNPs) which best splits the data into distinct homogeneous strata (e.g. those at high risk of asthma versus those at low risk of asthma). A conditional inference tree was built by first identifying the SNP (in this case, rs2243250 in IL4) which best discriminates asthma cases and non-allergic controls and implementing a binary split. Then the groups of children along each subsequent branch are treated as individual datasets and the regression analysis was repeated to identify the SNP that next best discriminates between asthma cases and controls. This process is repeated over and over for each of the resulting subsets until the stopping rules are met. We used the significance level of the conditional independence tests as α = 0.05 for our stopping criterion and the minimum number of children in a node considered for splitting was 200 for Caucasian and 80 for African American. The variable selection procedure and the stopping rules allow the application of statistical test procedures to minimize over-fitting issues [13]. Using the predict function, we then classed individuals as affected or unaffected based on the final tree, and used the predicted and actual disease status to calculate classification accuracy. In contrast to traditional diagnostic tests, such as cluster analysis [14], that typically classify patients into one of two groups, RP results identify several genetically characterized groups with associated asthma risk ranging from very low to very high.

We used Ingenuity Pathways Analysis (IPA) 8.6 (Ingenuity Systems, Mountain View, CA, USA), to demonstrate whether the RP interacting genes are part of an integrated and interconnected biological networks that involved in genes that have functional commonalities in both races. A data set containing RP gene identifiers was uploaded into IPA to map and generate putative networks based on the manually curated knowledge database of pathway interactions extracted from the literature. The gene network was generated using both direct and indirect relationships/connectivity. These networks were ranked by scores that measured the probability that the genes were included in the network by chance alone.

### SNP Imputation

For the replication Caucasian (GCC cases and CCC controls) and African American dbGaP (CAMP trio dataset) populations, we utilized the available genotyping data from the Affymetrix 6.0 SNP chip (http://www.ncbi.nlm.nih.gov/gap). However, none of the five IL4 SNPs evaluated in our Caucasian population and only two SNPs in African American populations were present on the Affymetrix® 6.0 SNP chip. Imputation was performed to infer genotypes at untyped markers using MACH 1.0.16 (http://www.sph.umich.edu/csg/MaCH), which uses a hidden Markov model to estimate an underlying set of unphased genotypes for each individual in a cohort. We used information on patterns of haplotype variation in the HapMap CEU and YRI samples (release 22) as our reference haplotype. We only considered SNPs that were either genotyped or could be imputed with relatively high quality (RSQ >0.4). The estimated mismatch rate in Markov model is about 0.001 for both populations.

For the Caucasian population, both imputed and genotyped SNPs were tested for association with asthma status using additive logistic regression models in PLINK. For the dbGaP CAMP dataset, association analysis was performed using the transmission disequilibrium test (TDT) described by Spielman and Ewens [15]. The TDT test evaluates the observed number of parent-offspring transmissions of alleles, compared with the number of transmissions expected by chance. Only parents heterozygous for the polymorphism tested are informative for the test. Association was tested using chi-square statistics. We applied imputation methods to validate our initial association and expands the test to untyped variants.

## Results

### Demographics of cases and controls

Basic descriptive statistics of the study populations by race is provided in Table 2. The mean age of Caucasian children was significantly less for asthmatic and allergic children compared to the non-allergic controls (p<0.0001). For African American children, there were significantly more males than females in the asthma group compared to both the allergic control group (p = 0.004) and non-allergic control group (p = 0.02). Therefore, associations between asthmatics and non-allergic children and between allergic children and non-allergic children were adjusted for age and gender in addition to population stratification.

**Table 2:** Sample size and covariates in both Caucasian and African American population.

Variable	Asthmatic Group		Allergic Group		Non-Allergic Controls	
	Caucasian	African Am.	Caucasian	African. Am.	Caucasian	African Am.
Total children (n)	420	330	269	150	298	51
Children after exclusions (n)^a^	413	315	261	147	298	51
Mean age (years)	10.1^b^	10.3	10.3^b^	10.8	12.0	11.4
Percent male	55.2%	63.2%^b^^,^ c	56.3%	48.3%	48.0%	45.1%

a Indicates the number children after children with missing call rates above 20% were removed.

b Indicates significant differences (p<0.05) with similar race non-allergic normal control children.

c Indicates significant differences (p<0.05) with similar race allergic children. African Am. indicates African American race.

### Allele frequencies vary by race

A comparison of allele frequency differences for 111 out of the 259 SNPs in Caucasian versus African Americans asthmatics (red) and Caucasian versus African American non-allergic control groups (blue) were statistically significant (Figure S1). The average absolute allele frequency difference in asthmatic allele frequency between Caucasian and African Americans was 0.129±0.107 with range from 0.0017 to 0.484. Non-allergic control children were more similar between Caucasian and African American groups than the asthmatic groups. Allele frequencies within the admixed African American populations are intermediate between the respective ancestral HapMap Phase 3 (European and African) populations (data not shown).

### Single SNP association, majority of SNP associations do not overlap between European ancestry and African American

**Figure 1:**
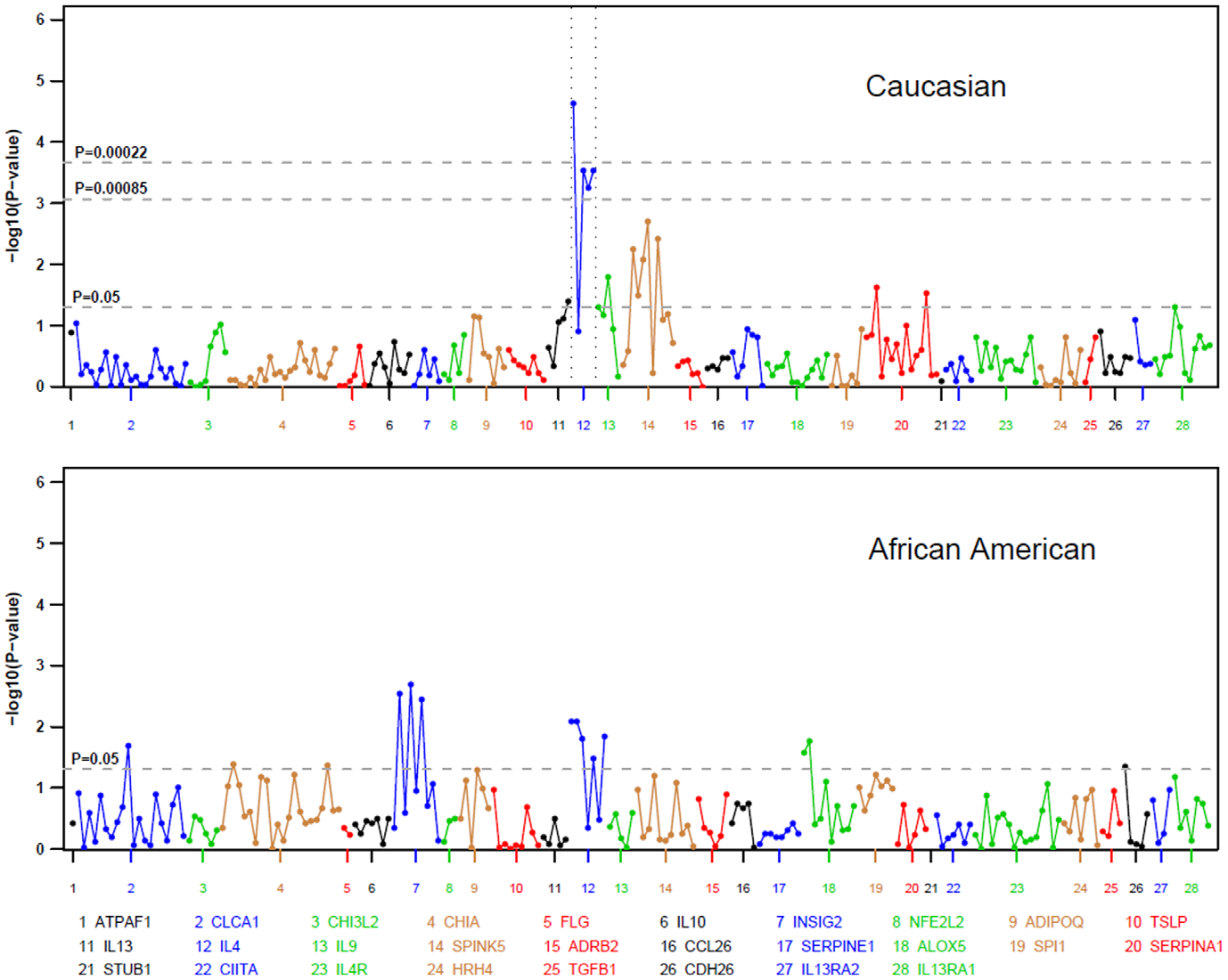
Associations between European and African Ancestry asthmatics vs. non-allergic controls. Associations between the 230 total SNPs within the 28 candidate genes were tested using the additive model after adjusting for age, gender and population stratification. The upper line corresponds to the conservative Bonferroni adjusted p value 0.00022. The middle line corresponds to the Bonferroni adjusted p value 0.00085 considering a LD correlation of 0.25. SNPs significant at this level (all in IL4) include rs2243250, rs243268, rs2243274 and rs43282. The lower line is a nominal significance p value = 0.05. SNPs are plotted on the x-axis according to their position on each candidate gene across the chromosome against association with asthma on the y axis (shown as log10 p value).

Among children with European ancestry, significant single SNP associations between asthmatics and non-allergic controls were detected in 13 of the 230 SNPs in 5 of the 28 genes (p-value = 0.05). These include SNPs in IL4, SPINK5, SERPINA1, IL9 and IL13 (Table S1). To take into account the LD correlation among SNPs, we used a modified Bonferroni adjustment cut-off [10] to determine the significance with a p-value of 0.00085. With this criterion, 4 SNPs in IL4 significantly increased the risk for asthma by approximately twofold (Table 3). In fact, IL4 rs2243250 remained significant even after the traditional and highly conservative Bonferroni adjustment (Figure 1). Considering the number of assays performed, IL4 showed an excess number of significant SNPs associated with asthma (4 out of 5), while a larger gene such as CLCA1, with 23 SNPs, showed no significant SNP associations.

**Table 3:** IL4 gene single locus association in Caucasian and African American population.

IL4		Asthmatics vs. Non-Allergic Controls							
		Caucasian				African American			
Frequency cases/controls =		413/298				315/51			
SNP (major/minor)	Function	MAF				MAF			
		Asthmatics	controls	OR	P-value	Asthmatics	controls	OR	P-value
rs2243250 (C/T)^a^	Promoter	0.195	0.116	2.00 (1.45,2.75)	0.00002	0.332	0.471	0.56 (0.37,0.86)	0.008
rs2243282 (C/A)	Intronic	0.180	0.117	1.81 (1.31,2.50)	0.0003	0.336	0.294	1.26 (0.79,2.02)	0.34
rs2243274 (G/A)^a^	Intronic	0.188	0.126	1.74 (1.27,2.38)	0.0006	0.389	0.500	0.64 (0.42, 0.96)	0.03
rs2243268 (A/C)	Intronic	0.178	0.116	1.81 (1.32,2.50)	0.0003	0.250	0.220	1.22 (0.73,2.06)	0.45
rs2243263 (G/C)	Intronic	0.113	0.135	0.77 (0.55,1.08)	0.13	0.167	0.265	0.54 (0.32,0.89)	0.016
rs2243248 (T/G)	Promoter					0.146	0.245	0.47 (0.27,0.82)	0.008
rs2243283 (C/G)	Intronic					0.152	0.039	4.4 (1.34,14.44)	0.015

Associations between asthmatic and non-allergic controls, and between allergic and non-allergic controls were tested independently and odds ratios (OR) were determined using logistic regression based on the minor allele after adjusting for age, gender and population stratification.

a Indicates major and minor alleles are reversed in African American children. Confidence intervals are indicated in parenthesis, MAF stands for minor allele frequency.

In the African American children, 14 SNPs in 6 genes were significantly associated with asthma (p-value = 0.05, Figure 1). These included SNPs in the IL4, INSIG2, CHIA, ALOX5, CLCA1 and CDH26 (Table S2). IL4 rs2243250 and rs2243274 were associated with asthma in both races (p-value <0.05). In the African American cohort, there were no significantly associated SNPs after Bonferroni adjustment (cutoff p-value of 0.0005). Interestingly, the minor allele frequency of these SNPs differs by race.

**Figure 2:**
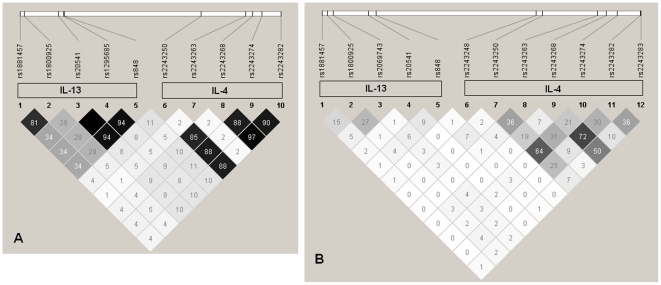
Pair-wise LD statistics. Pairs of common SNPs in genomic regions containing IL4 and IL13 in the Caucasian (A) and African American (B) population. The positions of SNPs within the IL4 and IL13 genes are shown above the plot. Values in boxes are r^2^ measures on a decimal scale (i.e. 97 represent r^2^ = 0.97), indicating extent of LD between two SNPs. Box without numbers have r^2^ = 1. The shade of each square indicates the strength of the LD relationship between pairs of SNPs.

To investigate if the strong single SNP association of IL4 gene is independent of IL13, linkage disequilibrium (LD) analyses were performed. IL13 is an adjacent cytokine gene, which lies 200 kb away from IL4 on chromosome 5q31 and has many structural and functional similarities with IL4 including a shared receptor (IL4Rα). All the genotyped IL4 SNPs in Caucasians and African Americans were studied for LD patterns. In both populations, LD within the IL4 gene was strong. However, LD was not observed between IL13 and IL4 in the African American population, while weak LD was observed between IL13 and IL4 genes in Caucasians (Figure 2). As LD is known to be highly influenced by ancestry, the observed patterns of LD and SNP relationships indicate that these populations are genetically different. There was no significant difference in LD pattern between cases and control subjects in either population (data not shown).

### Replication for IL4 in both races, and discovery of additional IL4 variants through imputation

Analysis of SNPs imputed from Affymetrix data revealed similar significant associations with asthma. In fact, for Caucasians, the effect sizes for the replication studies were greater than those observed in the discovery analyses. For example, the odds ratio of asthma for *IL4* promoter *SNP rs2243250* was 2.00 (95% CI 1.18–2.75) in our discovery analysis compared with the imputed GCC/CCC OR of 3.86 (95% CI 1.58–9.41). Further, additional SNPs were identified through imputation studies. Notably IL4 SNPs rs2227284 and rs2227282 increased the odds of asthma in Caucasian children by 5.2 (95% CI 2.68–10.0 p = 1.04E-06) (Table 3, Table 4). Although the replication control cohort was composed of adult asthmatic and non-asthmatic subjects, IL4 SNPs were significantly associated with asthma (Table 4). The replication of IL4 gene in children and adult cohorts implies its broader implication in asthma independent of development stages.

**Table 4:** IL4 gene imputation based association/replication in Caucasian and African American population.

IL4			Asthmatic (GCC) Vs. non-asthmatic controls (CCC)Caucasian		Childhood Asthma Management Program, CAMP (dbGaP)African American	
Frequency cases/controls =			74/211		42 trios	
	SNP ID	Function	OR*	P-value	OR**	P-value
Replication association	rs2243250	Promoter	3.86	0.003	2.15	0.019
	rs2243282	Intronic	3.86	0.003	1.3	0.53
	rs2243274	Intronic	2.97	0.0076	1.73	0.086
	rs2243268	Intronic	3.86	0.003	1.08	0.84
	rs2243263	Intronic			2.22	0.04
	rs2243248	Promoter			1.38	0.49
	rs2243283	Intronic			1	1
Discovery of untyped SNP association	rs2070874	Promoter	3.86	0.003		
	rs734244	Intron	3.86	0.003		
	rs2227284	Intron	5.17	1.04E-06		
	rs2227282	Intron	5.17	1.04E-06		
	rs2243266	Intron	3.86	0.003		
	rs2243267	Intron	3.86	0.003		
	rs2243288	Intron	2.97	0.0076		
	rs2243289	Intron	3.86	0.003		
	rs2243290	Intron	3.86	0.003		
	rs2243240	Promoter			0	0.0455
	rs2243246	Promoter			0.3571	0.03895
	rs2243252	Intron			0	0.0455

Similarly, in the African American population, the odds of asthma for IL4 SNP rs2243250 increased from 1.75 (95% CI 1.16–2.70) in the discovery analysis to 2.15 in the replication analysis (Table 3, Table 4). In addition, two IL4 promoter SNPs rs2243240 and rs2243246 discovered through imputation were also significantly associated with asthma (p-value <0.05) (Table 4).

### Gene- gene interactions and gene networks differ by race

To identify the SNP combination that best discriminates between asthma cases and non-allergic controls, we explored the gene-gene interactions (epistasis) among all 259 SNPs across the 28 candidate genes using RP.

**Figure 3:**
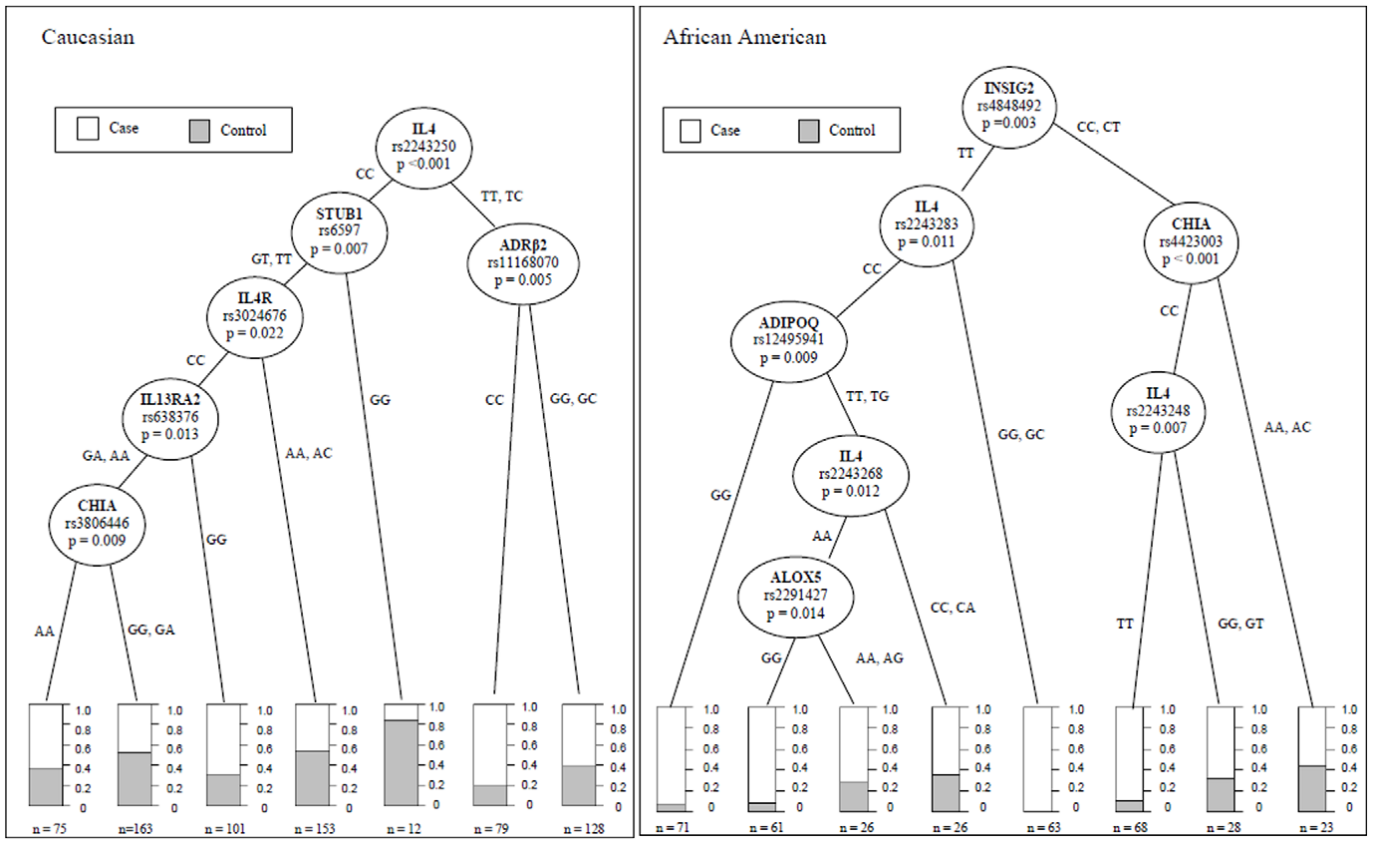
RP based gene-gene interactions of asthmatics vs. non-allergic controls. Using the program PARTY (implemented in R), non-parametric recursive partitioning was performed to identify combination of SNPs that together had the greatest ability to discriminate between asthmatic and non-allergic controls. All the 257 SNPs within the 28 candidate genes were evaluated in the process. For the stopping criterion we use the nominal level of the conditional independence test of α = 0.05. The final trees were enough to achieve 62% discrimination accuracy between the asthmatic and non-allergic control individuals for Caucasian population and 77% for African American population. The number of subgroup is indicated below each terminal node.

For the Caucasian population, a total of 6 SNPs from the total of 259 SNPs (in genes IL4, STUB1, ADRβ2, IL4Rα, IL13Rα2 and CHIA) remained in the final tree from the RP process (see Figure 3a). At the top of the tree, the most asthma predictive SNP was rs2243250, an extensively studied IL4 promoter variant [16], [17], [18], [19], [20], [21], [22], [23], [24], [25]. Interestingly, other SNPs were not significantly associated with asthma in univariate SNP association analysis, but appeared to be discriminative between asthmatic and non-allergic children in the multivariate model.

In the African American children, 5 SNPs in 5 genes together significantly discriminate between asthmatic and non-allergic children. INSIG2 rs4848492 was the most predictive gene following by IL4, CHIA, ADIPOQ and ALOX5 (see Figure 3b). Only two genes (IL4 and CHIA) were common in both races.

Ingenuity Pathways Analysis (IPA) demonstrated that RP based interacting genes are part of an interconnected gene network that involved in related biological activities and functional commonalities. In Caucasian, the most enriched IPA canonical pathways in the 6 genes (p<3.21*10^−4^) were IL4 signaling and T helper cell differentiation. In African Americans, airway inflammation in asthma and role of cytokines in mediating communication between immune cells were the most enriched pathways among the 5 genes (p<1.89*10^−2^). Gene ontology analysis of the African American network showed enrichment for specific biological functions, including arachidonate 5-lipoxygenase activity (p = 2.2×10^−6^), mevalonate kinase activity (p = 1.5×10^−3^) and interleukin-4 receptor binding (p = 1.5×10^−3^). Gene ontology analysis of the Caucasian based Network showed cytokine binding (p = 1.9×10^−12^), cytokine receptor activity (p = 1.4×10^−10^). And transmembrane receptor activity (p = 3.7×10^−6^). Enriched biological process includes production of molecular mediator involved in inflammatory response (p = 9.7×10^−9^) for AA and immune response (p = 1.0×10^−20^) for CEU.

## Discussion

To our knowledge, this is the largest candidate genes association study that has examined racial differences in childhood asthma. Through this systematic study, we have simultaneously studied both Caucasian and African American asthmatic children and demonstrated that these populations predominantly exhibit different patterns of association between genetic variants and asthma. To accomplish this goal we used well characterized European ancestry and African American children who live in the same geographic region of the greater Cincinnati area. Using both cohorts we have shown that only 1 of 28 genes had associations in both populations, as well as only 2 genes were common across the two races in the recursive partitioning analysis. Indeed, different gene networks were associated with asthma in children with European ancestry versus African Americans suggesting that there may be distinct mechanisms underlying the pathogenesis and expression of asthma in these 2 subgroups. Simultaneous investigation of risk variants across European and African American populations enabled the identification of population specific risk alleles and disease pathways, which may contribute to health disparity. The results from this study may also assist in fine-mapping of genetic associations by exploiting the differences in linkage disequilibrium between populations to narrow the range of marker alleles demarking regions that contain a true biologically relevant variant.

These analyses revealed two major findings. First, we confirmed the importance of IL4 genetic variation in the risk of pediatric asthma, and present evidence of replication among the African-American population. While IL4 has been consistently reported to be associated with asthma in Caucasian, Asian, and Hispanic populations, two of the four SNPs, which reached Bonferroni corrected significance in the Caucasian children (rs2243250 and rs2243274) replicated (p<0.05) in the African American children (Table 3). While non-coding IL4 rare variants have been associated with asthma susceptibility in African Americans [26], the association of these two SNPs is novel to this population. This result suggests that some common immunological mechanisms (at these variants) may underlie childhood asthma across different ethnic backgrounds. However, most studied SNPs showed no evidence of replication between Caucasian and African American children. For example, IL4 SNPs, which are highly significant in the Caucasian group such as rs2243282 and rs2243268 didn't reach 5% significance level in African American population. In contrast, SNP rs4448492 in the INSIG2 gene was associated (P = 0.002) with asthma in African American population. However, this SNP was not significant even without adjustment (at 5%) in Caucasian population. Several SNPs have shown different allele frequencies between the two races (Figure S1). This result suggests that these genes do not harbor susceptibility variants common to both races due to a) variation in signatures of natural selection resulting in differences in allele frequencies; b) varying linkage disequilibrium patterns at causal loci across different populations (as shown for IL4 – Figure 2); and/or (c) there may be common and distinct pathways that contribute to the development and expression of asthma phenotypes between these two groups. It also remains possible that we do not have sufficient statistical power with the current sample size to detect statistical significance, although this is unlikely for the observed lack of association of INSIG2 in the Caucasian subset. To determine the power of this to detect expected ORs for IL4 coding SNPs in both Caucasian and African American population, we conducted an ad-hoc analysis with the software Quanto [27]. With our sample size, we have 96% and 71% power to detect the association of rs2243250 with asthma in Caucasian and African ancestry population, respectively. This ad-hoc power analysis provides sufficient evidence that we have high power in Caucasian and moderate power in African American to detect true effects. The lack of SNP replication in these two populations emphasizes the need to consider ancestry background and detailed examination of population SNPs allele frequency across populations of different and mixed ancestry as well as non-genetic factors.

Secondly, Using RP, we report for the first time an interaction of six genes affecting European ancestry pediatric asthma: rs2243250 (IL4), rs6597 (STUB1) rs11168070 (ADRβ2), rs3024676 (IL4Rα), rs638376 (IL13Rα2) and rs3806446 (CHIA). These SNPs resulted in 62% accuracy of asthmatic and non-allergic classification. Similarly seven SNPs in five genes rs4848492 (INSIG2), rs2243283 (IL4), rs4423003 (CHIA), rs2243283 (IL4), rs12495941 (ADIPOQ), rs2243268 (IL4), and rs2291427 (ALOX5) in African American children had 77% discriminate power between asthmatic and non-allergic individuals. The combination of genotypes in these interactive SNPs can help to pin-point individuals with greater asthma risk (Figure 3). Importantly, the RP method may elucidate associations, which may be missed using single SNP association. For example, variants in STUB1 and ADRβ2 genes in Caucasian and variants in CHIA and ADIPOQ genes in African American were not associated with asthma in the single SNP analysis; however, in conditional inference framework taking rs2243250 and rs4848492 as a major discriminatory SNPs in Caucasian and African American respectively, variation in these genes is highly associated with asthma (p<0.01). Kabesch et al. [17] reported strong gene-gene interactions among genes involving Th3-cell differentiation and signaling pathways. Our study showed that using the RP approach, SNPs that are weakly or not associated in the univariate analysis could discriminate between asthma and non-allergic control individuals in both races. This finding clearly indicates that the effect of one gene may not be disclosed if the effect of another gene is not considered [28], suggesting that the true effect may be driven by gene-gene interaction, rather than by the main effect of each gene by itself.

**Figure 4:**
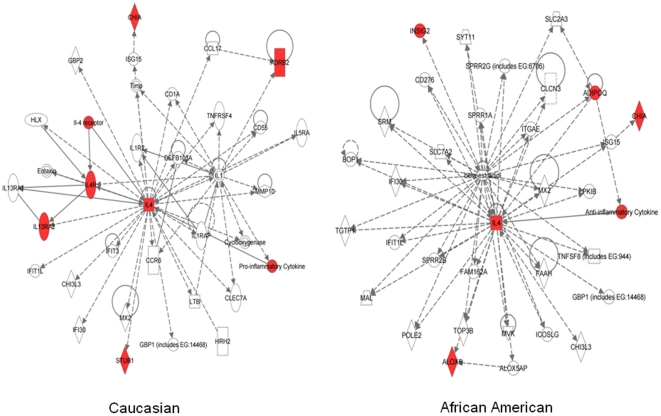
Ingenuity Pathway Analysis (IPA) Interactive network. IPA network for recursive partitioning prioritized genes. Genes with red node are focused genes in our analysis, others are generated through the network analysis from the Ingenuity Pathways Knowledge Base (http://www.ingenuity.com). Edges are displayed with labels that describe the nature of the relationship between the nodes. All edges are supported by at least one reference from the literature, or from canonical information stored in the Ingenuity Pathways Knowledge Base. Edges are displayed with labels that describe the nature of the relationship between the nodes. The lines between genes represent known interactions, with solid lines representing direct interactions and dashed lines representing indirect interactions. Nodes are displayed using various shapes that represent the functional class of the gene product. Nodes are displayed using various shapes that represent the functional class of the gene product (see legend).

Further analysis using Ingenuity Pathways Analysis (IPA) revealed that these RP based interactive genes belong to an interconnected and interactive gene network, indicating that they are involved in related biological activities and have functional commonalities (Figure 4a, b). We also used IPA to characterize the enrichment of specific pathway components into functionally differentiated gene groups [29]. The most enriched (p≤3*10^−4^) canonical pathway in Caucasian population was IL4 signaling whereas airway inflammation in asthma was the most enriched (p<1.36*10^−3^) pathway in African American (data not shown). Differences in the genetic architecture of individuals may have affected determinant pathways in different ways. However, both enriched IPA pathways in both races have essential roles in asthma pathogenesis [30]. In network analysis, IL4 was the major hub gene in both Caucasian and African American (Figure 4a, b). These results were not unexpected given that IL4 is a critical effector in the generation of allergic inflammation and IgE production, and is one of the most relevant genes in regulating the Th2 profile of allergic subjects [31]. IL4 is central to B cell heavy class switching from immunoglobulin M (IgM) to IgE, and to the maturation of T helper (Th) cells towards the Th2 phenotype [32]. One of the variants (rs2243250), which was most strongly associated with asthma, lies in the IL4 promoter region which has been implicated and replicated in more than 11 studies [17], [19], [20], [21], [22], [23], [25], [33], [34]. IL4 rs2243250 is a C-to-T mutation that lies upstream from the open reading frame of the gene. It has previously been shown to increase promoter activity of IL4 transcription and was associated with elevated levels of serum IgE in asthmatic families [25].

In critically evaluating our results, it is important to note that our analyses, and hence interpretations, are subject to several limitations. First, SNP allele frequencies and association were determined by using relatively small sample sizes (see Methods). However, it should be noted that large sample sizes may not help powering genetic studies and improve our understanding of the genetic underpinnings of allergy phenotypes as much as precise phenotyping [35]. In the present study we show that use of well-characterized control populations (see Methods) in genetic association studies can overcome relatively small sample sizes to identify risk variants. Further, in order to overcome the relatively small sample size in the AA cohort, we sought to replicate our findings using publically available datasets but found similarly small AA cohorts. Thus, there is a clear need for larger AA cohorts in future studies. Second, to reduce the chance of potential false positive results from multiple testing, we corrected the p-values using Bonferroni adjustment which accounted for the LD among SNPs. As the Bonferroni adjustment is notably conservative, the LD adjustment provides minimization of false positives. We believe that this approach provides a reasonable balance between type I and type II error. Nonetheless, it is likely that we are missing true associations, which may provide insight into racial differences and similarities. Third, the environmental influences between our case and control groups may be different, especially between adults and children. Fourth, our study showed a positive association, but it does not always imply causality. Hence, further studies are needed to confirm the findings and to identify functional variants causally linked to asthma risk. The present study has notable strengths. First, we were able to conduct the analyses separately in each race, and were therefore able to account for the differences in allele frequencies, disease prevalence, and linkage disequilibrium patterns between these subpopulations. Second, our study used a custom designed array that includes more coverage of candidate genes/SNPs of interest and the inclusion of ancestry informative markers (AIMs) to account hidden ethnic variations.

In summary, through our systematic and comprehensive screen of variants in asthmatic children who live in the same geographic region, we have demonstrated the importance of IL4 genetic variation in both Caucasians and African American. Variants found in populations of both African and European ancestry may represent more universally important genes to the disorder [36]. The replication of IL4 SNPs in African ancestry can also potentially aid in refining and fine mapping associations due to the unique short range LD in this ethnicity. The use of a population with short LD will result in the greatest localization success rate in distinguishing the causal SNP from its neighbors. Based on the overall lack of SNPs concordance in association between European and African American asthmatic children, we suspect that rare and/or population-specific risk alleles may explain some of the associations in asthma, pointing to genetic heterogeneity in susceptibility alleles. These results also underline the importance of understanding differences in biologic and genetic factors driving asthma in different ancestral populations. Future fine-mapping and deep sequencing studies are needed to determine whether or not other SNPs can be found associated in African Americans as well as to identify both common and/or rare risk-causing alleles in the associated regions.

## Supporting Information

Figure S1 Allele frequency differences (delta) between Caucasian and African American for asthma and non-allergic controls, respectively.
(TIF)

Table S1 Significant SNPs by gene in Caucasian population
(DOC)

Table S2 Significant SNPs by gene in African American population
(DOC)
