# Supplementary figures and images for: Differences in Candidate Gene Association between European Ancestry and African American Asthmatic Children
Source: PLoS One. 2011 Feb 28;6(2):e16522. doi: 10.1371/journal.pone.0016522 (PMC3046166; doi:10.1371/journal.pone.0016522)

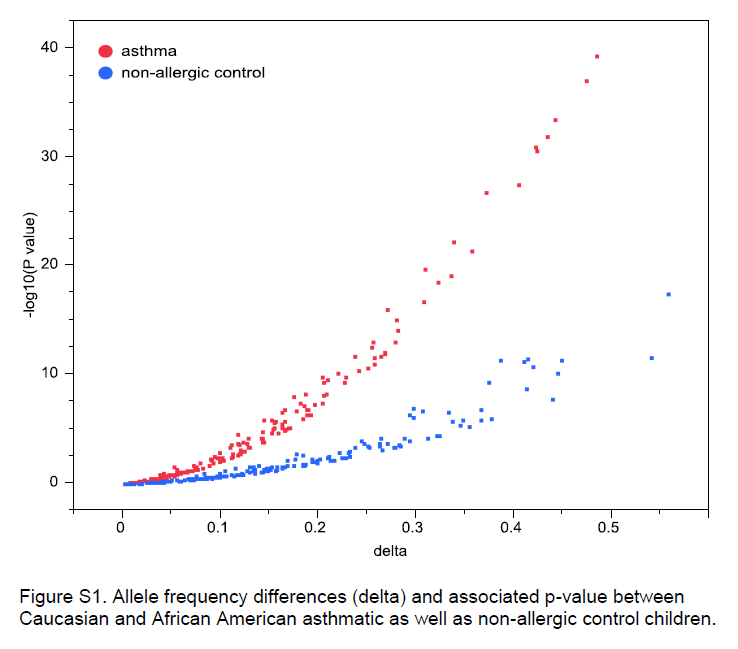

Supplement: Figure S1 — Allele frequency differences (delta) between Caucasian and African American for asthma and non-allergic controls, respectively. (TIF) [file pone.0016522.s001.tif]
