# Supplementary material for: Differences in Candidate Gene Association between European Ancestry and African American Asthmatic Children
Source: PLoS One. 2011 Feb 28;6(2):e16522. doi: 10.1371/journal.pone.0016522 (PMC3046166; doi:10.1371/journal.pone.0016522)
Supplement: Table S2 — Significant SNPs by gene in African American population (DOC) [file pone.0016522.s003.doc]

| Table S2. Significant SNPs by gene in African American population | | | | | | | | |
| --- | --- | --- | --- | --- | --- | --- | --- | --- |
|  | Asthmatic vs. Non-allergic controls | |  | Allergic vs. Non- allergic controls | |  | *Asthmatic vs. Allergic controls* | |
| Gene | Minimum  (P-value) | Significant SNPs from most significant to least significant | Gene | Minimum  (P-value) | Significant SNPs from most significant to least significant | Gene | Minimum  (P-value) | *Significant SNPs from most significant to least significant* |
| IL-4 | 0.008 | rs2243250 rs2243248 rs2243283 rs2243263  rs2243274 | IL-4 | 0.024 | rs2243283 rs2243263 | IL-4 | 0.0096 | *rs2243248* |
| INSIG2 | 0.002 | rs4848492 rs11123469 rs12613329 | ADIPOQ | 0.018 | rs12495941 | IL10 | 0.003 | *rs2222202* |
| CHIA | 0.041 | rs7411387 rs2820093 | CHIA | 0.048 | rs7411387 | IL-4Rα | 0.015 | *rs2074570 rs1805016* |
| ALOX5 | 0.017 | rs7919239 rs4986832 | TGFB1 | 0.045 | rs2241715 | ALOX5 | 0.001 | *rs7919239 rs4986832* |
| CLCA1 | 0.020 | rs3765989 | CLCA1 | 0.042 | rs3765989 | CLCA1 | 0.012 | *rs2753334* |
| CDH26 | 0.046 | rs6071043 | IL13RA2 | 0.041 | rs17095945 | IL13RA2 | 0.018 | *rs535036* |
|  |  |  | CIITA | 0.047 | rs1139564 | IL9 | 0.008 | *rs31564* |
|  |  |  |  |  |  | SERPINA1 | 0.024 | *rs8010121* |
|  |  |  |  |  |  | ADRB2 | 0.026 | *rs1042714* |
